# The Other Aortic Syndrome–Intramural Hematoma and Neurological Deficit: Case Report

**DOI:** 10.5811/cpcem.1421

**Published:** 2023-12-28

**Authors:** Laura E. Walker, Chris Marcellino, Bhargavi Gali

**Affiliations:** * Department of Emergency Medicine, Mayo Clinic, Rochester, Minnesota; † Department of Anesthesiology and Perioperative Medicine, Mayo Clinic, Rochester, Minnesota; ‡ Department of Neurologic Surgery, Mayo Clinic Health System Eau Claire, Eau Claire, Wisconsin; § Division of Intensive Care and Respiratory Therapy, Mayo Clinic, Rochester, Minnesota; ∥ Department of Anesthesiology and Perioperative Medicine, Mayo Clinic, Rochester, Minnesota

**Keywords:** *aortic syndromes*, *spinal cord ischemia*, *paraparesis*, *spinal drain*

## Abstract

**Introduction:**

Acute thoracic aortic syndromes are among the most concerning presentations in emergency medicine and are associated with significant morbidity and mortality. Thoracic aortic dissection is most common, followed by penetrating aortic ulcer and, least commonly, intramural hematoma.

**Case Report:**

A 67-year-old woman presented to the emergency department with chest and back pain, and sudden onset of paraparesis. Aortic intramural hematoma was diagnosed, and she underwent spinal drain placement with blood pressure control to optimize spinal cord perfusion.

**Discussion:**

When neurological deficits are present, rapid diagnosis of spinal ischemia and blood pressure optimization is vital. Spinal drains may be considered as an adjunctive treatment.

Population Health Research Capsule What do we already know about this clinical entity?

*Acute thoracic aortic syndromes are concerning presentations associated with significant morbidity and mortality.*

What makes this presentation of disease reportable?

*Emergency placement of a spinal drain to maximize spinal cord blood flow has not been reported for acute aortic syndrome presentations.*

What is the major learning point?

*Spinal drain placement to optimize blood flow to the spinal cord can be considered as adjunct treatment in cases of aortic syndrome to mitigate spinal cord ischemia.*

How might this improve emergency medicine practice?

*In cases of spinal cord ischemia associated with aortic syndromes, placement of a spinal drain is an opportunity to potentially improve long-term outcomes.*

## INTRODUCTION

Acute aortic syndromes are among the most concerning potential presentations in emergency medicine. The most common culprit of thoracic aortic syndromes is aortic dissection, followed by penetrating aortic ulcer and intramural hematoma. Incidences of each are 4.4, 2.1 and 1.2 per 100,000 person-years, respectively. Aortic dissection is associated with a higher rate of mortality, ranging between 1% risk of mortality per hour to 10–25% 30-day mortality rates. Intramural hematoma may progress to dissection and is also associated with a high risk of mortality.^1^ Commonly associated complications at presentation include pleural effusion, pericardial effusion and tamponade, periaortic effusion, aortic regurgitation, shock, stroke, cerebral malperfusion, visceral malperfusion, coronary ostial involvement, and renal impairment.^2^ Spinal cord ischemia is a rarer complication and can present with various deficits depending on the area of perfusion that is restricted.^3^

Typical management of aortic syndromes is focused on minimizing the risk of progression of dissection. This involves regulating blood pressure, with a goal systolic blood pressure of 100–120 millimeters of mercury (mm Hg).^4^ For situations in which there is concern for neurological ischemia, the balance between ensuring adequate perfusion and avoiding further vascular injury is tenuous.

## CASE REPORT

A 67-year-old woman presented to the emergency department (ED) via emergency medical services (EMS) after an initial call for chest pain. On initial assessment by EMS personnel, she complained of both chest pain and back pain that had been ongoing for several days. While en route to the ED she had sudden onset of paraparesis and increased emotional distress. There was no report of any recent trauma. Review of systems was otherwise negative.

Her initial exam revealed blood pressure of 165/99 mm Hg and otherwise normal vital signs. Pulses to all extremities were 2+, warm and well perfused. Strength was 5/5 in bilateral upper extremities, left lower extremity was 4/5 and right lower extremity was 0/5. Reflexes were normal in the upper extremities and on the left lower extremity but were absent on the right lower extremity. Initial laboratory studies including complete blood count, coagulation panel and basic metabolic panel were unremarkable, except for a mildly elevated high-sensitivity troponin of 23 nanograms per liter (ng/L) (reference range less than 12 ng/L), which did not have a significant change on repeat evaluation.

**Image 1.**
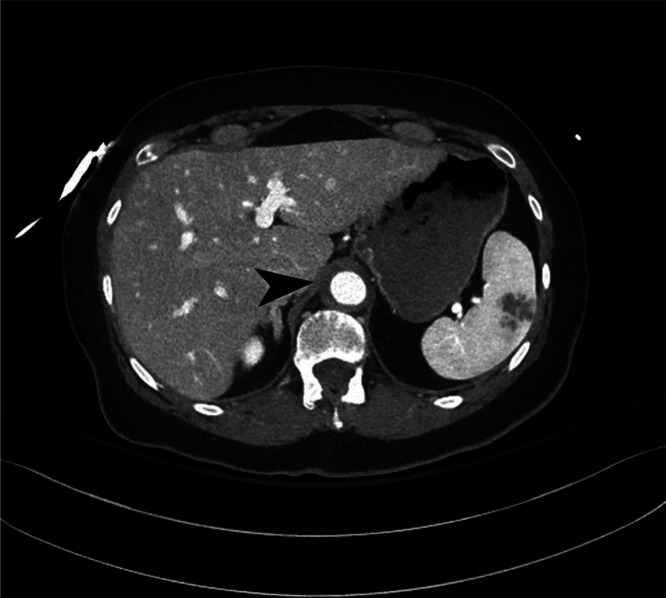
Demonstration of intramural thrombus of the thoracic aorta (arrow) in a patient with acute aortic syndrome, axial view.

**Image 2.**
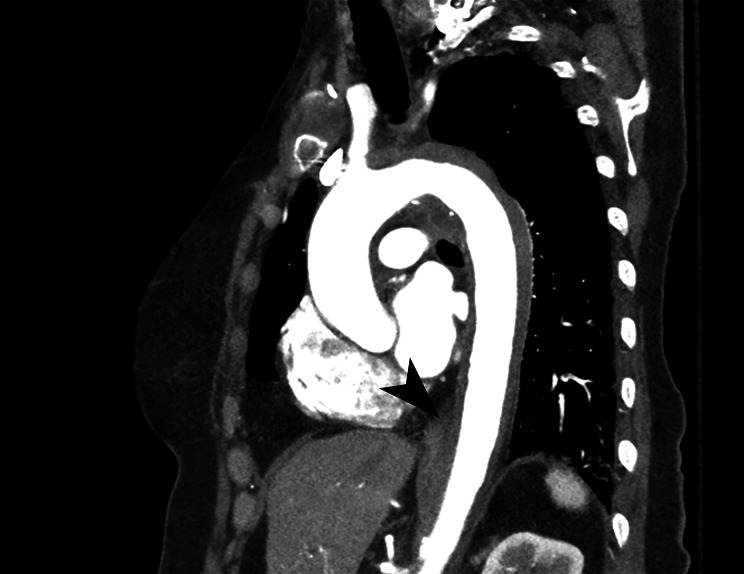
Demonstration of intramural thrombus of the thoracic aorta (arrow) in a patient with acute aortic syndrome, sagittal view.

Imaging studies of the chest, abdomen, pelvis, and spine were obtained. An initial portable chest radiograph identified prominence of the aorta, which was attributed to technique. Computed tomography (CT) angiography of the aorta revealed an intramural hematoma extending from just proximal to the left subclavian and extending into the abdomen and including the renal arteries (Images 1 and 2). No dissection flap was identified, but there was a potential ulceration described as “tiny” in the thoracic aorta. Spinal and brain CT imaging did not reveal any acute abnormalities.

Cardiothoracic and vascular surgeons were emergently consulted. Initial plans were developed for blood pressure control using clevidipine; however, after establishing concern for spinal cord ischemia (SCI) the recommended treatment was to maintain a goal mean arterial pressure (MAP) between 70–90 mm Hg to optimize spinal cord blood flow, as perfusion to the dominant segmental arteries was presumed to be impaired due to pressure from the hematoma.

The patient was rapidly admitted to the surgical critical care unit where a spinal drain was placed to reduce intrathecal pressure and promote perfusion from both the involved and uninvolved segmental arteries to the spinal cord by optimizing the perfusion gradient between systemic and intrathecal pressure. After drain placement, the patient had mild improvement in her weakness of the left lower extremity but persistent weakness of the right lower extremity. Cerebral spinal fluid (CSF) drainage was maintained for approximately 48 hours and weaned without neurological worsening. During her intensive care unit course she had additional improvement in right lower extremity strength. She did require clevidipine early in her course and norepinephrine infusions during sleep to keep MAP within the 70–90 mm Hg range. Use of both antihypertensive and pressors enabled the team to strike a balance between the risk of uncontrolled hypertension causing further intravascular hematoma formation and providing optimal perfusion to the spinal cord during the period of critical collateral formation. At the time of discharge from the hospital the patient had persistent, right-sided lower extremity weakness, but was able to perform activities of daily living with assistive devices.

## DISCUSSION

Spinal cord ischemia is a rare finding that can lead to permanent disability including paraplegia. Most cases of SCI or infarction are reported periprocedurally, commonly with thoracoabdominal aortic aneurysm repair.^5^ Spontaneous causes of SCI include aortic intramural hematoma, as in our patient, as well as other arterial occlusions resulting from arteriosclerosis, vasculitis, infection, embolic occlusion, thrombosis, and hypoperfusion of the spinal cord.^5^

**Figure 1.**
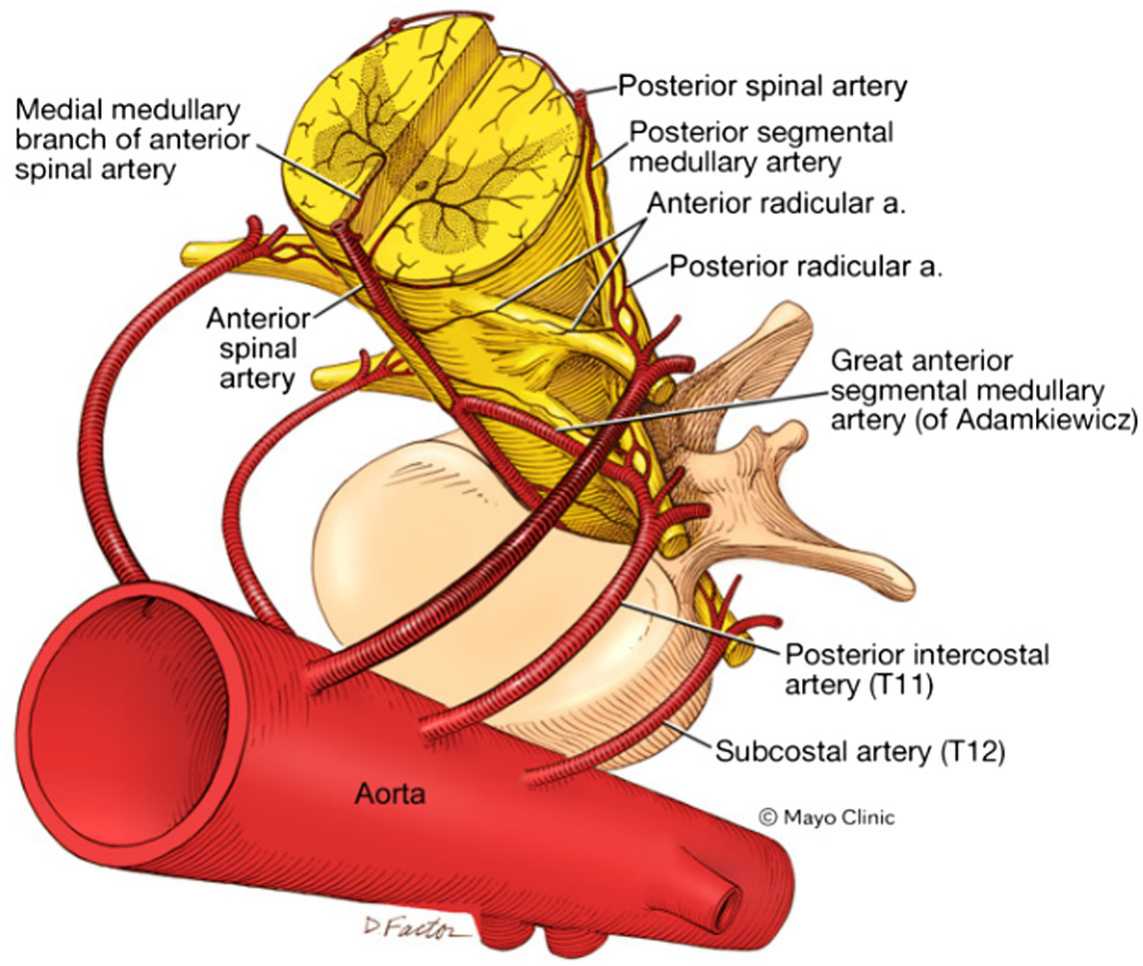
Diagram of the vascular supply to the spinal cord. An oblique diagram of the interface of the segmental vascular supply of the spinal cord from the intercostal and subcostal arteries as radicular branches to the longitudinal arteries of the spinal cord. The great anterior segmental artery (of Ademkiewicz) is the superior-most segmental artery in the diagram on the left anatomical side, which in most individuals anastomoses between the ninth and twelfth thoracic vertebrae, although occasionally it arises from a lumbar segment or on the right. In acute aortic syndromes, Spibal cord ischemia (SCI) typically is caused by injury to multiple segmental arteries, or the great anterior segmental artery alone (often in iatrogenic SCI.) Used with permission of Mayo Foundation for Medical Education and Research, all rights reserved. *a*, artery; *SCI*, spinal cord ischemia; *T11*, eleventh thoracic vertebra level; *T12*, twelfth thoracic vertebra level.

**Figure 2.**
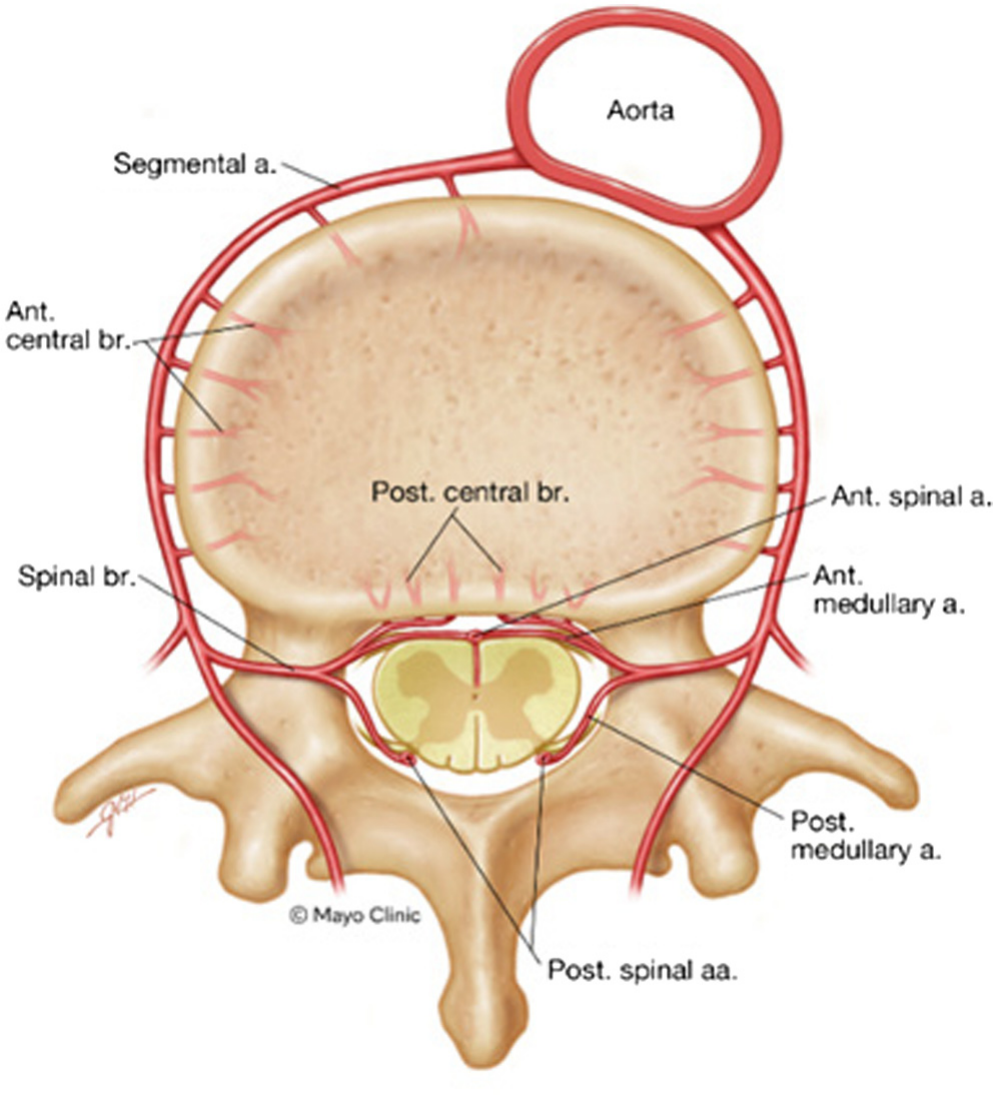
Cross-sectional diagram of a single segmental vascular physiological anastomosis to the spinal cord. A segmental artery of the aorta supplies collateral circulation to the longitudinal spinal arteries of the spinal cord and branches to bone and other supporting musculature and ligaments of the spine. Used with permission of Mayo Foundation for Medical Education and Research, all rights reserved. *a*, artery; *aa*, arteries; *ant*, anterior; *br*, branch; *post*, posterior.

The spinal cord is supplied proximally by three main longitudinal arteries: the anterior spinal artery (ASA); and two posterior spinal arteries.^6^ The ASA supplies approximately two-thirds of the spinal cord, and its diameter changes throughout its course. The narrowest segment of the ASA is in the thoracic region. Numerous segmental arteries provide collateral supply and typically arise from the vertebral and intercostal arteries. The thoracic spinal cord is most dependent on the collateral supply from these arteries, which typically enter from the left neural foramen. The great anterior segmental artery (of Ademkiewicz) is the most prominent radicular artery, between the ninth and twelfth thoracic vertebrae in most individuals (Figures 1 and 2).

The thoracic spinal cord receives arterial supply from the narrowest section of the ASA and is highly dependent on collaterals, thus most likely to suffer from SCI or infarction. Optimizing arterial supply to the spinal cord is the focus of periprocedural management of patients undergoing thoracic aortic repair and reduces the risk of spinal cord ischemia.^7^ This intervention is less commonly reported in acute treatment of aortic syndromes with SCI. One method involves increasing the MAP to increase spinal cord perfusion pressure (as spinal cord perfusion pressure gradient is equal to MAP minus intrathecal pressure). The second method, which our patient experienced, aims to control cerebrospinal perfusion pressure by use of a lumbar drain or intermittent draws of CSF to lower intrathecal pressure and improve perfusion. Complications of drain placement include intracranial hemorrhage, meningitis, spinal epidural hematoma, and subdural hematoma.^5^ Complications are reported in 1–4% of cases, with a recent retrospective, single-center study reporting a complication rate of 6.4%.^8^

## CONCLUSION

Our patient developed SCI from acute intramural hematoma and presented with chest and back pain. She did have persistent deficits related to SCI; however, her symptoms improved with placement of the spinal drain and elevation of the spinal cord perfusion pressure gradient. Using this treatment modality is different from typical management of aortic syndromes in the ED. The acute focus pivots from maintaining a reduced MAP to providing adequate pressure to perfuse the spinal cord. Our patient needed both pressors and antihypertensives during the acute phase of her presentation and treatment and was provided control of intrathecal pressure to ensure optimal spinal cord perfusion. Rapid diagnosis of SCI provides an opportunity to mitigate morbidity and should be considered when acute aortic syndromes are in the differential, accompanied by neurological deficits in the setting of normal extremity perfusion.
